# An Instance Segmentation Model Based on Deep Learning for Intelligent Diagnosis of Uterine Myomas in MRI

**DOI:** 10.3390/diagnostics13091525

**Published:** 2023-04-24

**Authors:** Haixia Pan, Meng Zhang, Wenpei Bai, Bin Li, Hongqiang Wang, Haotian Geng, Xiaoran Zhao, Dongdong Zhang, Yanan Li, Minghuang Chen

**Affiliations:** 1 College of Software, Beihang University, Beijing 100191, China; 2 Department of Obstetrics and Gynecology, Beijing Shijitan Hospital, Capital Medical University, Beijing 100038, China; 3 Department of MRI, Beijing Shijitan Hospital, Capital Medical University, Beijing 100038, China

**Keywords:** deep learning, instance segmentation, uterine myomas, magnetic resonance imaging (MRI), computer-aided diagnostics

## Abstract

Uterine myomas affect 70% of women of reproductive age, potentially impacting their fertility and health. Manual film reading is commonly used to identify uterine myomas, but it is time-consuming, laborious, and subjective. Clinical treatment requires the consideration of the positional relationship among the uterine wall, uterine cavity, and uterine myomas. However, due to their complex and variable shapes, the low contrast of adjacent tissues or organs, and indistinguishable edges, accurately identifying them in MRI is difficult. Our work addresses these challenges by proposing an instance segmentation network capable of automatically outputting the location, category, and masks of each organ and lesion. Specifically, we designed a new backbone that facilitates learning the shape features of object diversity, and filters out background noise interference. We optimized the anchor box generation strategy to provide better priors in order to enhance the process of bounding box prediction and regression. An adaptive iterative subdivision strategy ensures that the mask boundary details of objects are more realistic and accurate. We conducted extensive experiments to validate our network, which achieved better average precision (AP) results than those of state-of-the-art instance segmentation models. Compared to the baseline network, our model improved AP on the uterine wall, uterine cavity, and myomas by 8.8%, 8.4%, and 3.2%, respectively. Our work is the first to realize multiclass instance segmentation in uterine MRI, providing a convenient and objective reference for the clinical development of appropriate surgical plans, and has significant value in improving diagnostic efficiency and realizing the automatic auxiliary diagnosis of uterine myomas.

## 1. Introduction

Uterine myomas, also known as uterine leiomyomas, fibroids, or leiomyomas, are the most commonly encountered benign uterine tumors [1]. They have an incidence rate of 40–60% in women under 30 years old, and 70–80% in women over 50 years old [2]. Uterine myomas are responsible for 2–3% of women’s infertility [3] and are globally the most common indication for hysterectomy. In the United States, more than 479,000 hysterectomies are performed each year, with 46.6% due to myomas, and 47.7% occurring in women between the ages of 18 and 44 [4]. Uterine myomas can be single or multiple, varying in size, and have great heterogeneity in pathophysiology, size, location, and clinical symptoms [5]. The most common symptom is heavy menstrual bleeding (HMB), which often leads to anemia, fatigue, or dysmenorrhea [5,6,7]. Other possible symptoms are back pain and pelvic compression or pain, which can affect the quality of life. When uterine myomas exceed a certain size, they can put pressure on the bladder or intestines, causing bladder dysfunction or constipation, among other symptoms. In addition, uterine myomas may affect the outcome of pregnancy, and become the cause of infertility and recurrent abortion. Almost one-third of women with uterine myomas seek treatment [8].

The International Federation of Gynecology and Obstetrics (FIGO) classifies uterine myomas into eight types on the basis of their relationship to the uterine wall, uterine cavity, and mixed myomas [9]. This classification plays a crucial role in helping doctors in developing surgical plans. However, patient satisfaction with the current treatment plans is often low, leading to women undergoing major surgery such as hysterectomy [10]. Personalized treatments according to FIGO classification, main symptoms (HMB, infertility), and patients’ real intentions are necessary. Intelligent diagnosis is a significant current research highlight in the medical field [11,12,13], but there is a relative gap in the area of the auxiliary diagnosis of uterine diseases. Therefore, it is urgent and necessary to perform auxiliary diagnostic research on the uterine region, which could significantly benefit patients with uterine myomas and gynecologists.

Several methods have been proposed for segmenting the uterus. Yao et al. [14] used the cascade method of the fast-marching and Laplacian level sets to segment the uterus. Liao et al. [15] proposed an adaptive local region and edge-based active contour model to segment uterine myomas in ultrasound images. Militello et al. [16] discussed the study of magnetic resonance-guided focused ultrasound (MRgFUS) in the treatment of uterine myomas. Casarino et al. [17] proposed a region-growth-based method that could segment myomas with different pixel intensity levels. Fallahi et al. [18] proposed a fuzzy C-means-based method to segment uterine myomas in T1-weighted MR-enhanced images. The MR-guided high-intensity focused ultrasound was used by Antila et al. [19] to segment the uterine myoma region. Militello et al. [20] proposed a two-dimensional segmentation method for uterine myomas in MRgFUS treatment evaluation using fuzzy C-means and adaptive threshold segmentation methods. However, accurate segmentation results cannot be obtained without clear gray boundary differences, especially in scenes with complex and diverse shapes of tissues or organs. Deep-learning technology can automate the entire process of medical image segmentation and reduce dependence on expert intervention. Hodneland et al. [21] used a 3D segmentation model to automatically segment endometrial cancer on MRI, and Kurata et al. first tried to use UNet to automatically segment the uterus on MRI [22]. Zhang et al. [23] proposed HIFUNet for the segmentation of the uterus, myomas, and the spine before HIFU surgery. Niu et al. [24] used the Hessian matrix to extract image edges and completed the semantic segmentation of uterine MRI. Tang et al. [25] proposed AR-UNet for the automatic segmentation of uterine myomas from T2-weighted MRI.

**Figure 1:**
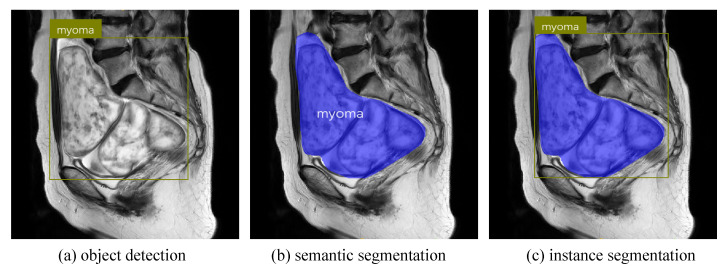
Visualization of visual tasks. (**a**) Object detection; (**b**) semantic segmentation; (**c**) instance segmentation.

Most existing studies on the uterine region employ traditional or machine-learning methods, with some deep-learning studies being limited to semantic segmentation of uterine myomas or uterus. These studies only achieved pixel-level classification in images and could not distinguish between different instances of the same class. Instance segmentation combines the advantages of object detection and semantic segmentation by achieving pixel-level classification, and object positioning and classification (as shown in Figure 1). It has the ability to accurately determine boundaries, size, and category of human organs or lesions while understanding multiangle and indepth semantic information. Instance segmentation can be divided into two- and one-stage methods. Two-stage models generally achieve higher segmentation AP, but have longer segmentation times. Representative methods include Mask-RCNN [26], RefineMask [27], and SSAP [28]. One-stage models can achieve faster segmentation than two-stage models can, but their AP is generally lower. Typical models include YOLACT++ [29] and SOLOv2 [30]. The Mask-RCNN model is a two-stage instance segmentation model proposed by He et al. It mainly improves the ROIAlign operation on the basis of high-precision object detection model Faster-RCNN [31], and added a mask branch to predict segmentation masks, achieving 37.1% AP in the COCO dataset [32]. Since the introduction of Mask-RCNN, its excellent performance and model design ideas have become benchmarks for many subsequent instance segmentation models. Although many new models have good innovative ideas and new architectures, their metrics often cannot reach or exceed those of Mask-RCNN [29,33,34,35].

The instance segmentation of myomas, and the uterine wall and cavity in MR images is an essential precondition for achieving FIGO classification and preoperative evaluation. To the best of our knowledge, no relevant instance segmentation studies have been reported [36]. The main challenges are as follows: (1) large variations in shape and size between categories; (2) the low contrast between adjacent organs and tissues, hindering distinguishing boundaries; (3) difficulty in identifying fine and narrow uterine-cavity and small-scale myomas. As precision is more important than real-time performance in the medical field, we optimized and improved the Mask-RCNN model, which could segment the uterine wall, uterine cavity, and myomas in sagittal (SAG) T2W MR images. The main contributions of this paper are summarized as follows:

- We propose an instance segmentation network that could achieve the full automatic instance identification of multiple classes within the uterine region.
- We designed a new backbone network that reduces the loss of feature information caused by continuous convolutional operations, and can better adapt to complex and variable object shapes, and resist noise.
- We optimized the generation method of anchor boxes. We used the k-means algorithm to adjust the size and scale of anchor boxes of each feature layer. This approach reduces the generation of redundant anchor boxes and accelerates bounding box regression.
- We introduce a fine segmentation mask head. In the mask branch, we used an iterative subdivision strategy to gradually refine rough masks and correct any misclassified pixels.
- We validated our approach with some excellent models and visualized its segmentation performance.

The structure of this paper is as follows: Section 2 describes the dataset and the proposed network architecture. Section 3 covers the experimental configuration and results, and evaluation metrics. In Section 4, we analyze and discuss the experimental results. Lastly, Section 5 provides conclusions and perspectives.

## 2. Materials and Methods

**Figure 2:**
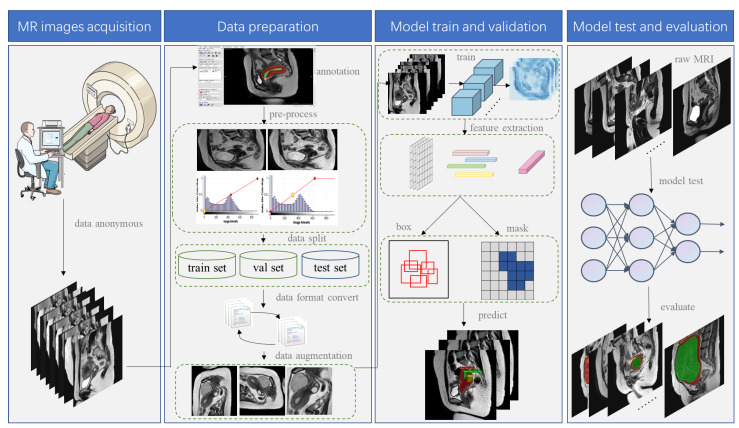
Study flowchart.

The flowchart of this study is shown in Figure 2, including dataset acquisition and preprocessing, and the design, training, and testing of the instance segmentation model.

### 2.1. Dataset Description

MR imaging visualizes the size, location, and shape of myomas better than ultrasound and CT imaging do.It has irreplaceable advantages in determining the relationship between myomas and the uterine cavity, and showing the presence or absence of comorbid uterine pathologies elsewhere [37]. T2W imaging is the primary examination sequence for pelvic diseases, as it clearly displays the anatomical structure of the uterus. The SAG section is the ideal orientation to show a panoramic view of the uterus, displaying the uterine contour well and providing an intuitive anatomical basis for the protruding direction of uterine myomas.

#### 2.1.1. Image Acquisition

We included SAG T2W MR images from 143 patients with uterine myomas who had undergone pelvic MR scanning at Beijing Shijitan Hospital from January 2015 to August 2022 with an age range of 21–86 years. The MR images were acquired using a 3.0T PHILIPS INGENIA ultrahigh-field MR imaging system. Each MR volume contained slices with pixel dimensions ranging from 512 × 512 to 880 × 880, slice thickness ranging from 4 to 6 mm, and a slice spacing of 10% of the slice thickness. The MRI scan parameters are shown in Table 1. To protect patient privacy, all private patient information is anonymous in MRI.

#### 2.1.2. Image Preparation and Preprocessing

**Figure 3:**
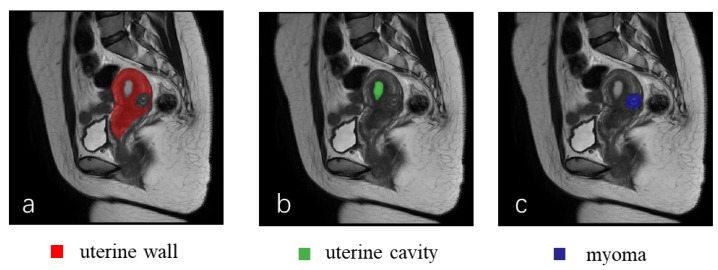
Visualization of annotation results. (**a**) Uterine wall; (**b**) uterine cavity; (**c**) myomas.

**Data Annotation.** The dataset was annotated by four doctors with intermediate professional titles, and three imaging physicians as reviewers. The annotators used medical image annotation tool ITK-SNAP (www.itksnap.org, accessed on 11 June 2022) to annotate the polygonal contours in the categories of the uterine wall, uterine cavity, and uterine myomas. The final annotation results were confirmed by reviewing physicians, and any unqualified annotations were returned for revision until final approval. The annotated visualization results are shown in Figure 3.

**Data Preprocessing.** MR images are characterized by low contrast, offset fields, and blurred boundaries between tissues that require image preprocessing. We performed the following preprocessing operations: (1) Contrast adjustment: Uterine myomas display low signal intensity in raw MRI, resulting in relatively dark images with poor differentiation between lesions, especially between myomas and the uterine wall. Therefore, we applied an adaptive histogram equalization operation to each image. (2) Normalization: we normalized MR image intensities into the same range using the Z score. (3) Offset field correction: MR images are subject to offset field interference during the imaging process, leading to different gray values for the same organ tissue in the image. To correct this, we used the N4ITK [38] offset field correction method.

**Data Split.** For the supervised deep-learning task, we evaluated the model performance using three dataset settings. We used manually annotated MR images as the ground truth (GT) and randomly divided the patient dataset into training, validation, and test sets in an 8:1:1 ratio. The statistical results for the number of images and instances are shown in Table 2.

**Figure 4:**
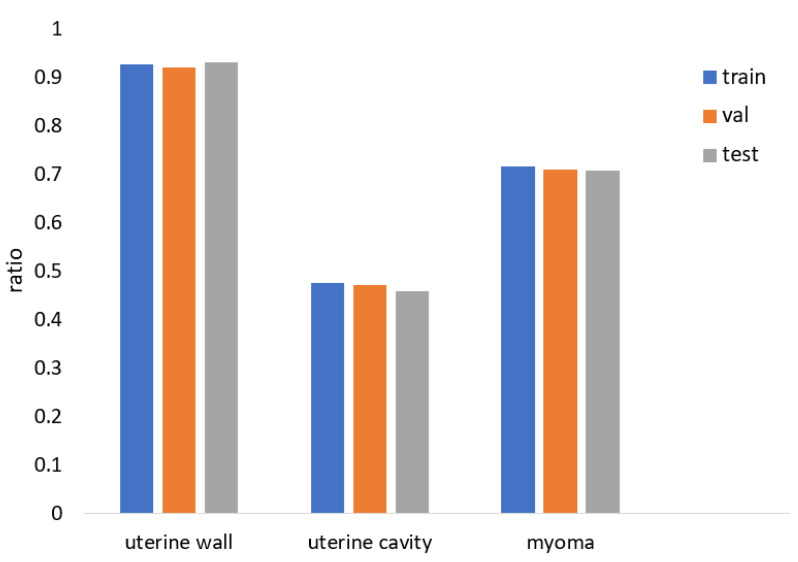
Multiclass data sample distribution statistics.

Moreover, we conducted a quantitative analysis of the data samples for the three categories in the dataset as shown in Figure 4. The samples of each category maintained the same distribution in the divided dataset, which indicates that the method of dividing the dataset was reasonable. However, the samples for the uterine-cavity category were very few, indicating an overall issue with sample imbalance. We provide corresponding solutions in Section 3.2.

**Figure 5:**
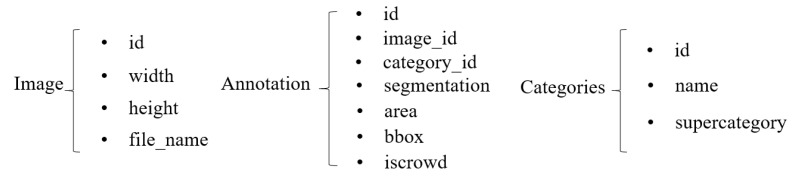
COCO dataset format.

**COCO dataset format conversion.** Instance segmentation models typically use the COCO dataset annotation format [32], which consists of three parts: basic image information, annotation information, and classification information (as shown in Figure 5). To facilitate data processing and model training, we converted our data into the COCO format.

### 2.2. Instance Segmentation Approach for the Uterine Region

**Figure 6:**
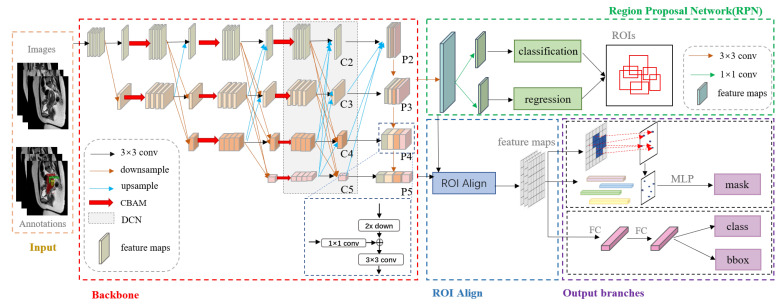
The network architecture of instance segmentation. First, preprocessed images and corresponding label files are fed into the network during training, while only images are used during the testing phase. The backbone network then extracts multiscale feature maps that are input into the RPN, which performs ROIAlign operations. Lastly, three branches generate predictions for categories, bounding box positions, and image masks.

The overall network structure is shown in Figure 6, consisting of three main stages: (1) Feature extraction and feature fusion: the backbone performs feature extraction on the input medical images, allowing for detail and semantic features to complement each other for feature enhancement. (2) Region proposal network (RPN): multiscale feature maps output from the backbone are input into the RPN to obtain high-quality regions of interest (ROIs). ROI Align was then performed for feature extraction to improve subsequent localization and classification accuracy. (3) Prediction: multiple prediction heads predict and output the categories, locations, and masks of the focus area in the medical images.

#### 2.2.1. Feature Extraction and Fusion

The MR images that we had acquired had complex backgrounds, varying-size and -shape myomas, and unclear edge contours, requiring a neural network with strong feature-extraction ability. Therefore, we used HRNetv2p for high-resolution feature extraction and multiscale feature fusion [39]. We first used 3 × 3 convolution to downsample the feature map to its original 1/4 size, and then performed continuous convolution for feature extraction, obtaining parallel high- and low-resolution branches. The output of each stage was obtained from the repetitive exchange of information from multibranch feature maps. Feature fusion was then performed on the multiscale feature maps, so that each output retained certain details while obtaining semantic information. The low-resolution layer used bilinear interpolation to upsample the high-resolution layer and concatenate the obtained feature representations. Lastly, multiscale features were obtained by using average pooling step by step.

Regular convolutional kernels are usually fixed squares, resulting in similar receptive fields for objects of different shapes and sizes in the same feature layer. The fixed position sampling of the convolutional kernel hinders adaptively extracting the actual shape features of objects, and limits the fitting ability, leading to missed pixel points. Regular convolution is defined as follows:

(1) $$y\left(p_{0}\right)=\sum_{p_{l}\in K}\omega \left(p_{l}\right)F\left(p_{0}+p_{l}\right)$$

where, $p_{l}$ represents the local position of convolution K,ω represents the weight, $p_{0}$ represents the center of the convolutional kernel, and F(·) represents the activation function of the convolution.

To better adapt to the complex and diverse shapes of the objects, we introduced deformable convolution (DCN) [40] into the backbone, which is defined as follows:

(2) $$y\left(p_{0}\right)=\sum_{p_{l}\in K}\omega \left(p_{l}\right)F\left(p_{0}+p_{l}+\Delta p_{ab}\right)$$

**Figure 7:**
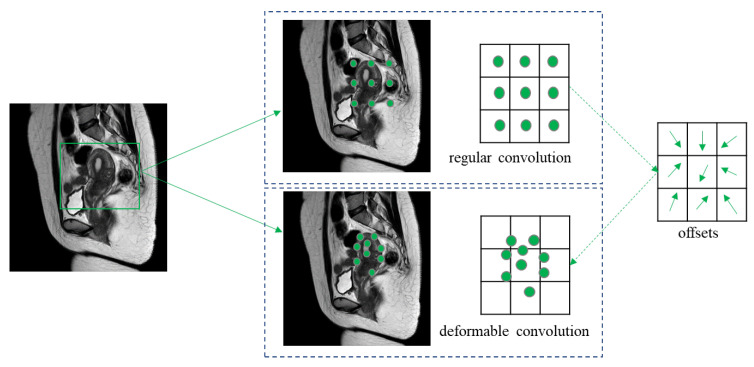
Comparison of regular and deformable convolution.

The introduction of offsets $△p_{ab}$ in convolutional kernels enables random sampling around the current sampling point, expanding the receptive field beyond the previous regular square, as shown in Figure 7. This approach helps in alleviating segmentation difficulties caused by serious losses of shape detail information. Learning offset variables only requires a few additional parameters and calculations.

**Figure 8:**
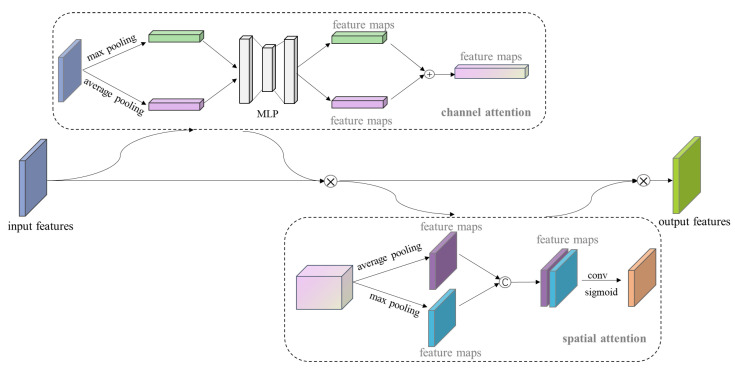
CBAM structure.

The compact positioning of the uterine wall, uterine cavity, and myomas hinders accurately recognizing and identifying these objects. To address this issue, we added a convolutional block attention module (CBAM) [41] to the model whose structure is shown in Figure 8. The CBAM module is a lightweight attention mechanism that enhances the representational power of the model by selectively highlighting the most relevant features and suppressing irrelevant ones. The module consists of two types of attention blocks: the channel attention block (CAB) and the spatial attention block (SAB). First, the CBAM module takes in a feature map F(H × W × C) and passes it through the CAM module, which performs global average pooling and max pooling in parallel. Two sets of 1 × 1 × C feature maps are obtained and jointly input into an MLP with two layers of neurons. The MLP uses element-wise addition for feature fusion, and applies the sigmoid activation function to obtain the feature weight value Mc (F) of each channel in the input feature layer. This weight value is then applied to the input feature map to enhance channel attention. Next, the output of the CAM module is fed into the SAM module, which performs average pooling and max pooling to obtain two sets of H × W × 1 feature maps. These feature maps are fused using channel concatenation, and the feature weight Ms (F) for the spatial dimension is obtained after activation using the sigmoid function. This weight is applied to the input feature map to achieve attention weighting on both the channel and spatial dimensions.

**Figure 9:**
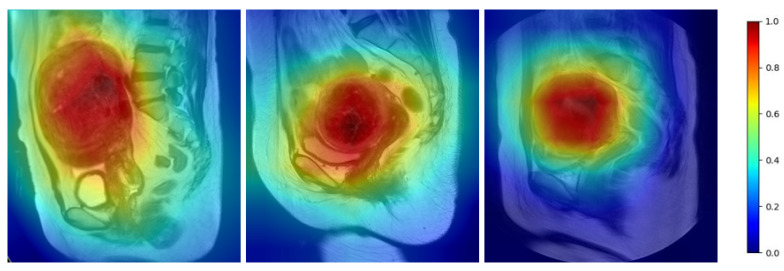
Heat map of the uterine region.

The MR image contains both the uterine region and other organs such as the spine and bowel that may have similar signal intensity. To demonstrate the feature extraction ability of the model, we present a heat map in Figure 9. The darker red in the heat map indicates higher activation intensity that received more attention from the network, while blue indicates weaker activation intensity and corresponds to irrelevant regions. The heat map shows that the uterus in different positions and with different sizes was sufficiently activated, while irrelevant information in the image was suppressed.

#### 2.2.2. Anchor Box Generation Strategy

After the multiscale feature maps had been generated, they were input into the region proposal network (RPN), which traverses each pixel on the feature map to generate anchor boxes. These boxes serve as references for subsequent classification and box regression. Anchor boxes have various sizes and aspect ratios to cover objects of different sizes. However, using incorrectly sized anchor boxes can increase the training time, affect positional regression, and impact the segmentation within the boxes.

**Figure 10:**
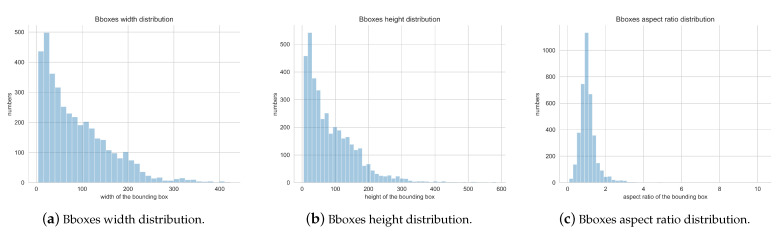
Box width, height, and aspect-ratio distributions.

To provide an appropriate anchor box size, we first computed the width, height, and aspect ratio of the object boxes in the dataset. As shown in Figure 10a,b, the width and height of the boxes were mostly within 260 × 260. Figure 10c shows that the aspect ratio was mostly less than 2, and when the aspect ratio was 1, the number of boxes was the largest.

We then used the k-means clustering algorithm to generate new anchor box sizes that better fit the objects in the uterine region. In this paper, we used the intersection over the union (IoU) to measure the distance between the samples and clustering centers, as shown in Equation (3).

(3) $$\begin{array}{c} D(box,centroid)=1-IoU(box,centroid) \end{array}$$

where *D* is the required distance for calculation, the box is not the anchor selection information of the cluster center, and the centroid is the anchor selection information of the cluster center.

**Figure 11:**
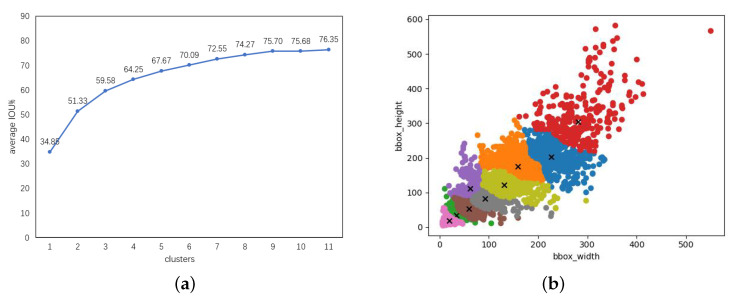
Bounding-box clustering results. (**a**) Average IoU under different cluster centers. (**b**) Visualization of the clustering effect of 9 clustering centers.

Figure 11a illustrates the relationship between different clusters and the average IoU. As the number of clusters increased, the average IoU also increased, and the slope of the curve was significantly flattened when the clusters were more than 9. When k = 9, the average IoU reached 75.7%, which was almost the maximum among the 11 clusters. Increasing the number of clusters generated more anchor boxes, which significantly increased the training time. Considering both IoU and computational efficiency, we chose k = 9 as the final number of clusters. Figure 11b shows the clustering effect of the boxes at that cluster size.

The output anchor box size was (17,16), (28,28), (50,45), (74,75), (103,167), (112,100), (142,137), (189,171), (235,255). After sorting from small to large, each group of three anchor sizes was applied to the small, medium-sized, and large feature maps that had been output by the feature extraction stage. Different levels of anchor scales could cover the effective receptive field range of each feature map. This ensures that each feature layer contains matching anchor boxes, which improves the subsequent box regression results. Specifically, P3 belonged to the shallow layer and had a small receptive field, rendering it suitable for predicting small objects, so the anchor should be smaller; P4 belonged to the middle layer and could predict medium-sized objects; P5 belonged to the deepest layer and had the largest receptive field, rendering it suitable for predicting large-scale objects, so the anchor was larger.

#### 2.2.3. Mask Branch

The traditional mask branch utilizes an encoder–decoder structure for dense prediction on a uniform grid, which can result in coarse mask details and indistinguishable edge regions that are not suitable for tasks requiring high edge accuracy. To improve the smoothness and clarity of mask boundaries, we used the PointRend module [42] to replace the traditional upsampling process. The PointRend module consists of three stages: point selection, point-level feature representation, and PointHead prediction. The first stage selects points that can be adaptively focused on indistinguishable boundary features in the image. During training, kN (*k* > 1) points are randomly selected from the feature map, and the most uncertain βN (β∈[0,1]) points are selected from them. During testing, an iterative coarse-to-fine strategy was adopted to render and refine the mask. Coarse prediction was performed on the low-level feature map, which contained more contextual and semantic information. After using bilinear interpolation for upsampling, the regular grid became denser, and the most uncertain points n${}_{i}^{*}$(i = 1, 2, 3, …, *N*) with a confidence level less than 0.5 were selected as the pixel points for correction. The selection method for these points is as follows:

(4) $$\begin{array}{c} n_{i}^{*}=\arg\min_{n_{i}}\left|p\left(n_{i}\right)-0.5\right| \end{array}$$

where $p(n_{i})$ is the probability for point $n_{i}$ to belong to the binary mask, and $n_{i}^{*}$ is the selected point.

**Figure 12:**
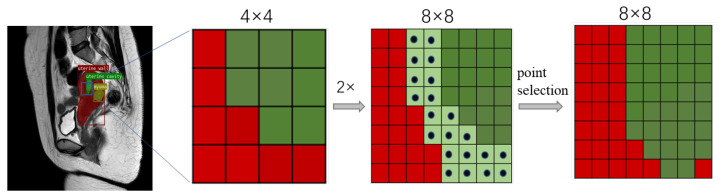
Adaptive iterative subdivision step of edge points on a uterine MR image. First, rough predictions are performed on a 4 × 4 grid, and bilinear interpolation is used to upsample twiceon the prediction. After that, the 21 most ambiguous points are selected on a finer, 8 × 8 grid. After PointHead’s prediction, detailed pointwise features are recovered. This process is repeated until the segmentation is upsampled to the desired spatial resolution.

The specific iterative subdivision process is shown in Figure 12. Through continuous iterative refinement, fuzzy edge points can be classified more clearly and accurately. Pointwise feature representation consists of combining fine-grained and high-level semantic features. PointHead is a few-parameter multilayer perceptron (MLP) with 3 hidden layers and 256 channels.

#### 2.2.4. Loss Function

The proposed network in this paper is a multitask network with a loss function consisting of three components: classification, object detection, and segmentation. We used weighting factors to balance the losses of each branch as shown in Equation (5):

(5) $$\begin{array}{c} L_{loss}=\lambda_{1}L_{cls}+\lambda_{2}L_{bbox}+\lambda_{3}L_{mask} \end{array}$$

where $\lambda_{i}$ (i = 1, 2, 3) is the weighting factor of each branch. After the experiment, $\lambda_{1}$ and $\lambda_{3}$ were set to 1, and $\lambda_{2}$ was set to 1.2.

$L_{cls}$ represents the classification loss that was calculated using the cross entropy loss function as shown in Equation (6). $L_{bbox}$ represents the bounding box localization and regression loss, as shown in Equation (7); the smooth L1 loss function was calculated as shown in Equation (8). $L_{mask}$ was composed of the loss generated by CoarseMaskHead and MaskPointHead, and it was calculated using the binary cross-entropy loss as shown in Equation (9).

(6) $$\begin{array}{c} L_{cls}=\frac{1}{N_{cls}}\sum_{i}L_{cls}\left(P_{i},P_{i}^{\prime }\right) \end{array}$$

(7) $$\begin{array}{c} L_{bbox}=\frac{1}{N_{reg}}\sum_{i}P_{i}^{\prime }L_{reg}\left(ti,ti^{\prime }\right) \end{array}$$

(8) $$\begin{array}{c} L_{reg}\left(ti,ti^{\prime }\right)=smoothL1\left(ti-ti^{\prime }\right) \end{array}$$

(9) $$\begin{array}{c} L_{mask}=-ylogy^{\prime }-\left(1-y\right)log\left(1-y^{\prime }\right) \end{array}$$

## 3. Experiments and Results

### 3.1. Evaluation Metrics

The COCO evaluation metric is the most widely used criterion for instance segmentation tasks. It uses AP to calculate the average precision and measure the performance of all classes. AP is defined using the IoU criterion, which measures the overlap between prediction masks and GT masks. In Table 3, ”area” refers to the number of pixels in the masks. Generally, a higher AP value indicates better results and is used as the final overall criterion. Since instance segmentation involves both detection and segmentation tasks, boxAP is used to represent the precision of the bounding box, and maskAP is used to indicate the precision of the mask.

### 3.2. Implementation Details

The experiments were conducted using PyTorch on an Ubuntu 20.04 operating system. We used an NVIDIA GeForce RTX 3060 (14 GB memory) with CUDA 10.2 and CuDNN with 7.6.3 for experiments. The SGD optimizer was used with an initial learning rate of 0.001, and momentum and weight decay settings of 0.9 and 0.0001, respectively; the model was trained for 60 epochs. The training process took approximately 6.5 h. The input image was resized to 512 × 512, and the batch size was set to 4. To balance the data samples, we applied class weighting by calculating the inverse frequency of each class in the training set. We assigned higher weights to the minority classes (uterine cavity), and lower weights to the majority class (uterine wall and myoma). This approach ensured that the model focused on the under-represented classes and avoided bias towards the majority class. We also used data augmentation techniques to increase the diversity and variability of the data, and reduce overfitting, including random rotation, central cropping, and vertical and horizontal flipping. All experiments in this study used the same dataset and experimental configuration.

### 3.3. Ablation Study

We conducted ablation experiments to evaluate the role of each structure and component in the model with the designed backbone structure (HRAD), the improved anchor box generation strategy of RPN (RA), and the PointRend module in the mask branch (PR). Table 4 shows that the HRAD structure played a significant role in improving the performance of the model, with an overall AP improvement of 3.7%. Improvements in RA and PR are also evident. By combining these structures and components, the AP improvement was 10.1% compared to the baseline model. These results demonstrate that HRAD achieved excellent feature extraction, providing a solid foundation for bounding box localization and segmentation masks.

The RA and PR structures are modifications of the detection branch and the mask branch, respectively. To evaluate their respective effects on bounding boxes and masks, we assessed them using the boxAP and maskAP metrics as shown in Table 5. An improvement in RA directly affected the localization and regression of bounding boxes, resulting in an increase of 0.9%, 1.4%, and 2.2% in small, medium-sized, and large boxAP scales, respectively. This improvement also affected the segmentation masks inside the boxes, leading to a certain improvement in maskAP, which is very promising. The improvement in PR mainly affected maskAP, as the method primarily enhanced the mask edges and largely left bounding box positions unaffected. Lastly, the combined effect of these two methods led to an improvement in the overall metrics.

**Figure 13:**
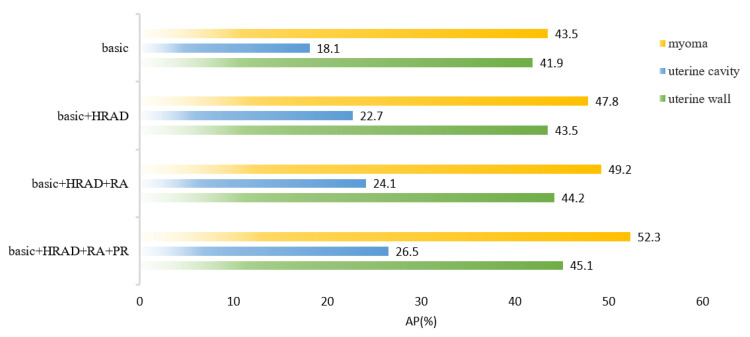
Performance of maskAP for each model structure and component in the uterine region.

Figure 13 visualizes the effect of each structure on the uterine wall, uterine cavity, and uterine myomas using bar charts. Comparing our proposed model with the basic model, we observed significant improvements of 8.8% and 8.4% in the uterine wall and uterine cavity, respectively, and of 3.2% in uterine myomas. Overall, these results demonstrate the effectiveness of each structure and component applied to uterine MRI segmentation.

**Figure 14:**
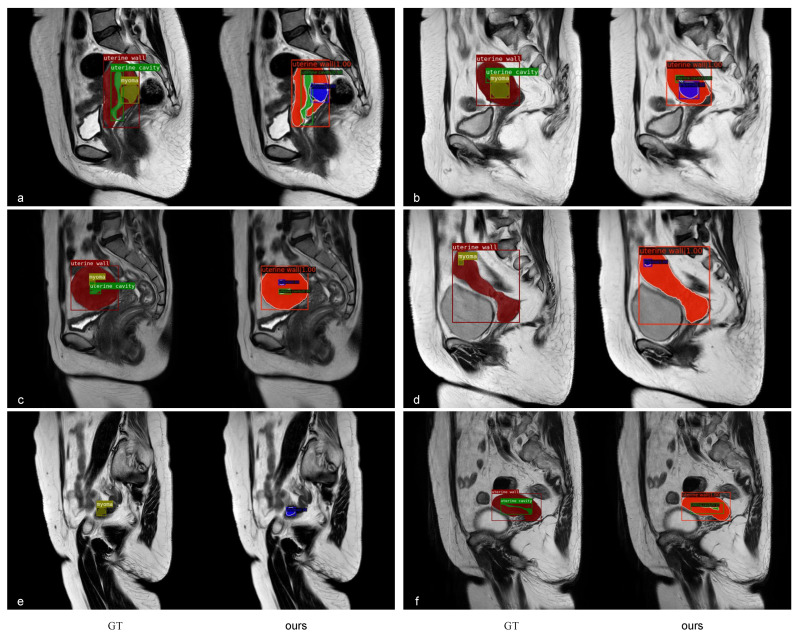
Visualization of the instancesegmentation results of our model in the uterine region. The left-hand side of each group represents GT masks, and the right-hand side displays the inference masks generated by our model. In GT masks, red represents the uterine wall, green represents the uterine cavity, and yellow represents myomas. In the masks of model inference, blue represents myomas, and the others were consistent with GT. The score on the bounding box is the predicted confidence value.

Figure 14 demonstrates the instance segmentation results of our model in the uterine region. To facilitate visual observation and description, we covered the masks on the raw MR image. Figure 14a–c show segmentation masks for multiclass coexistence; Figure 14d,e illustrate the masks of small myomas; Figure 14f shows the masks of the slender uterine cavity.

**Figure 15:**
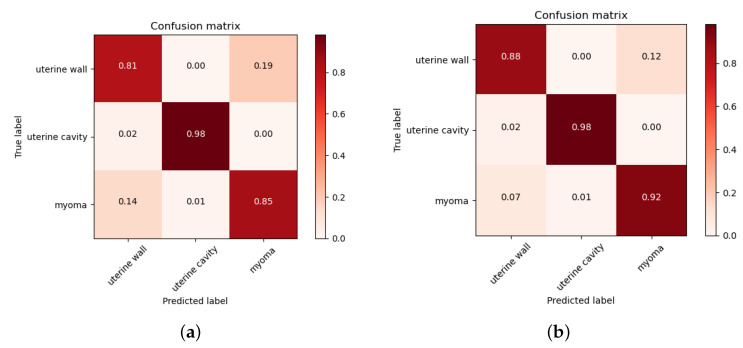
Confusion matrix of the test dataset. (**a**) Confusion matrix of the baseline model. (**b**) Confusion matrix of the proposed model.

Figure 15 shows the confusion matrix of the test dataset after model inference, which indicates whether the predicted category labels matched the true categories. The diagonal values represent the probability of each category being classified correctly, while off-diagonal values indicate prediction errors. Due to the high signal intensity and overall brightness of the uterine cavity, while both the uterine wall and myomas had low signal intensity and overall darkness, the uterine cavity was rarely predicted incorrectly, while the uterine wall and myomas were often confused. Comparing Figure 15a,b show that our model significantly improved prediction accuracy for the uterine wall and uterine myomas.

### 3.4. Comparison with Popular Models

We conducted comparative experiments with instance segmentation models that have been highly precise and strongly competitive in recent years, and the results are shown in Table 6. Our proposed model outperformed the other models in all metrics. Among the existing instance segmentation models, the Mask-RCNN network remained highly competitive. Our model improved upon this approach with significant gains, such as a 10.1% improvement in AP, a 4.6% improvement in AP${}_{s}$, a 5.7% improvement in AP${}_{m}$, and a 6.5% improvement in AP${}_{l}$.

**Figure 16:**
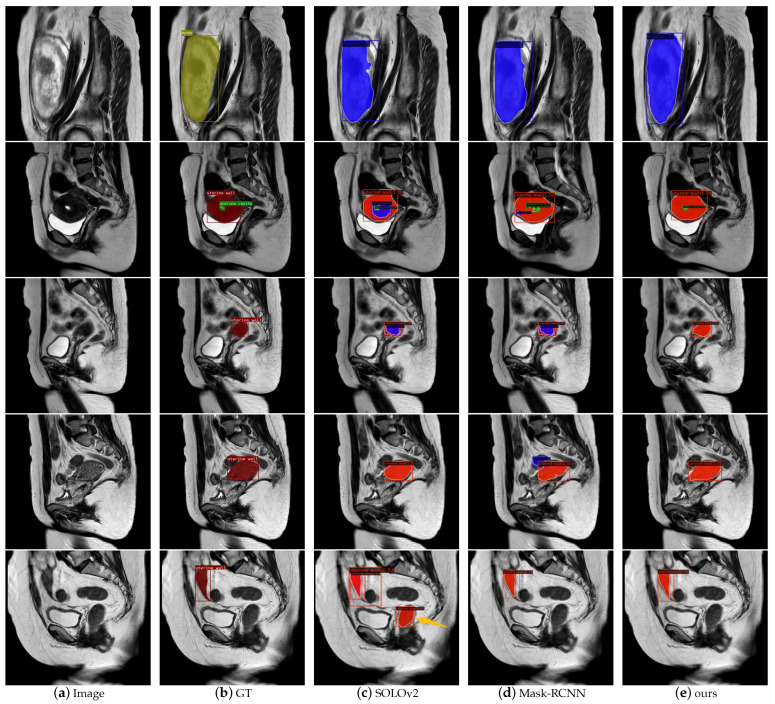
Segmentation visualization of different instance segmentation models in uterine region. In GT masks, red represents the uterine wall, green represents the uterine cavity, and yellow represents myomas. In the masks generated by the models, blue means myomas, and the others are consistent with GT.

We compared the visualization results of our approach with those of competitive models SOLOv2 and Mask-RCNN. Figure 16 shows several typical instance segmentation results in the uterine region. In the first row, it is evident that the segmentation mask of our model was closer to the shape and edge of the real myoma. This was attributed to the DCN and PointRend structures, which gave the model better deformable feature-learning and mask-refinement abilities. Rows 2 and 3 show that the other models misinterpreted myomas in the uterus due to the uneven signal intensity in the uterine wall. Rows 4 and 5 show that there were many organs and tissues in the MR image that had very similar signal intensities to those of the uterine region, causing the model to confuse them with our target. Overall, our model was more robust to noise, and the segmentation of edge details was smoother and more realistic.

**Figure 17:**
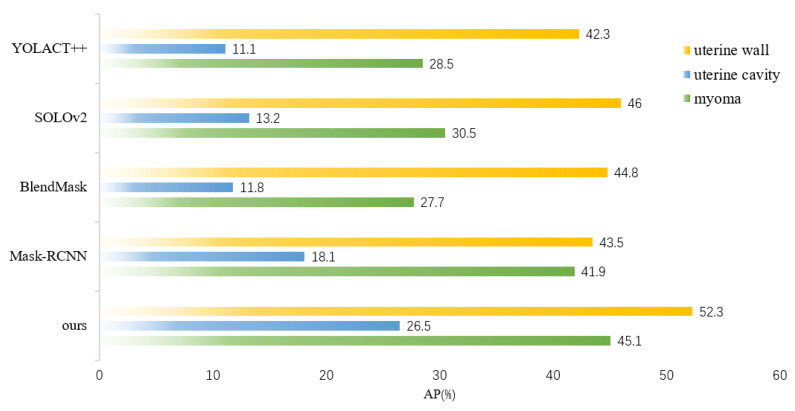
maskAP comparison of different instance segmentation models in the uterine region.

Figure 17 shows the maskAP results for multiple classes in the uterine region for each instance segmentation model. Our model outperformed the other models in terms of the uterine wall, uterine cavity, and myomas.

## 4. Discussion

In this paper, we proposed an instance segmentation model based on deep learning for the auxiliary diagnosis of uterine myomas in MRI. Our method achieved better AP results than those of state-of-the-art instance segmentation models on the same dataset. The visualization results demonstrate that the mask output of our method fit better with the real object. Specifically, in the uterine MRI with complex backgrounds, our model had better resistance to background noise and did not detect nonuterine objects as our targets. This is mainly because our backbone structure maintained high-resolution features containing detailed information, and the attention mechanism enhanced the focus on features related to the uterine region while filtering out irrelevant noise. Additionally, our improved anchor box generation strategy rendered our model more suitable for the size of multiple categories in the uterine region and could perform better at the small, medium-sized, and large scales. The DCN could learn the shape features of objects more flexibly, while the PointRend module further ensured the fineness of the mask for complex objects with various shapes.

However, the AP of the uterine cavity and the APs of all categories were relatively low, as shown in these metrics, mainly because uterine walls or myomas compress the uterine cavity, rendering it very thin and narrow, and there were some small-scale myomas in early onset or in different MRI slices. These objects had only a few pixels, hindering the model from learning useful features. In the future, we plan to conduct further research to address these issues and improve the results of these objects. Furthermore, the 3D image features of the uterus are essential in clinical and deep-learning technique research, as it can provide more contextual information and spatial features. Due to the limitation of GPU resources, we only conducted experiments on 2D images. Our next step is to extend computing resources, and explore the potential of instance segmentation on 3D uterine MR images.

## 5. Conclusions

In this paper, we proposed a deep-learning-based instance-segmentation model that could automatically output the class, location, and masks of the uterine wall, uterine cavity, and uterine myomas. Experimental verification and visualization results demonstrate that our approach had excellent instance segmentation ability in the uterine region. Our approach could reduce the burden of the manual segmentation of lesions for doctors, alleviate the pressure of manual film reading, accelerate the diagnostic process for uterine myomas, and improve patient satisfaction. It can also be used for the auxiliary diagnosis of uterine myomas, providing gynecologists with a quick and objective reference to develop individualized treatment plans, such as hysteroscopic and laparoscopic surgeries, and drug therapy. Relatively few studies use deep-learning technology to achieve instance segmentation in the uterine region, and this study provides a promising solution, and has potential applications in the diagnosis of uterine diseases. In the future, we will build larger and richer datasets, and strive to improve the segmentation precision of our model on the uterine cavity and small-scale objects to further enhance the application of instance segmentation techniques in medical-image-assisted diagnosis. 

## Figures and Tables

**Table 1:** Scan parameters of MR images.

Variable	Value
Repetition time (TR)	4200 ms
Echo time (TE)	130 ms
Field of view (FOV)	24 × 24 cm
Voxel	0.8 × 0.8 × 4.0 cm${}^{3}$
Reverse angle	90°
Age (year)	50.04 ± 11.37 *

* Age is the mean value ± S.D.

**Table 2:** Number of images and instances in the dataset.

Dataset	Number of Images	Number of Instances
Training	1349	2825
Validation	168	344
Test	170	351
Total	1687	3520

**Table 3:** Evaluation metrics of the COCO dataset.

Metrics	Means
AP	IoU = 0.50:0.05:0.95
AP${}_{50}$	IoU = 0.50
AP${}_{75}$	IoU = 0.75
AP${}_{s}$	area < 32${}^{2}$
AP${}_{m}$	32${}^{2}$ < area < 96${}^{2}$
AP${}_{l}$	area > 96${}^{2}$

**Table 4:** Performance evaluation of each network structure and component, ✓: adds corresponding improvements to the network.

HRAD	RA	PR	AP%	AP${}_{50}\%$	AP${}_{75}\%$
			34.5	56.8	30.0
✓			38.2	60.5	36.2
✓	✓		39.8	61.7	37.5
✓		✓	42.5	62.8	40.8
✓	✓	✓	44.6	64.7	41.3

**Table 5:** Performance evaluation of improved detection branch and mask branch. ✓: adds corresponding improvements to the network.

RA	PR	boxAP			maskAP		
		AP${}_{s}\%$	AP${}_{m}\%$	AP${}_{l}\%$	AP${}_{s}\%$	AP${}_{m}\%$	AP${}_{l}\%$
		27.1	52.5	67.4	20.9	50.4	66.5
✓		28.0	53.9	69.6	21.5	51.7	67.3
	✓	26.8	52.7	67.7	21.8	52.2	68.8
✓	✓	28.5	54.4	67.8	22.3	53.4	69.0

**Table 6:** Performance comparison of different instance segmentation models.

Model	AP%	AP${}_{50}\%$	AP${}_{75}\%$	AP${}_{s}\%$	AP${}_{m}\%$	AP${}_{l}\%$
YOLACT++ [29]	27.3	47.0	21.7	11.1	25.7	59.1
SOLOv2 [30]	29.9	52.7	27.9	12.9	37.3	66.8
BlendMask [33]	28.1	45.3	23.8	11.7	27.6	57.2
E2EC [34]	30.8	55.7	29.6	13.0	41.4	68.7
Mask-RCNN [26]	34.5	56.8	30.0	17.7	47.7	62.5
ours	44.6	64.7	41.3	22.3	53.4	69.0

## Data Availability

Data are available upon reasonable request.
